# Death

**Published:** 1864-03

**Authors:** 


### DEATH.

DEATH is the cessation of life. When by a wound, concussion, or mental shock, the action of the heart is destroyed, the brain ceases to live at once, because life-giving blood ceases to be sent to the brain and it dies, as a fish dies without water. It is desirable to know in all cases that death has certainly taken place, to avoid the horrible fate of being buried alive, which perhaps has not occurred a dozen times since the world began; perhaps not once, unless by deliberate design, as a murder or execution. The credulous Fontenelle, who died a hundred years old in 1757, gathered from all history only a hundred cases, without any proof of their truthfulness. It is true that persons disinterred have been found turned over in their coffins, their grave-clothes disarranged and even torn. Sounds have come from coffins while being let down into the grave or soon after, but no authenticated account has ever come to the writer's notice of a person coming to life after the coffin has been screwed down; and yet coffins have been found burst open, and appearances have been observed which would naturally be exhibited after some desperate struggle. But it is the nature of all dead bodies to swell; this process commences on the instant of life's cessation, because decomposition begins preparatory to the corruption which precedes our return to that dust from which we came. This decomposition generates gases, which keep on expanding until they compel an outlet. There is a well-authenticated case, (and various similar instances,) where a body, after being laid on the dissecting-table, was suddenly heaved up and thrown on the floor in the presence of the young medical students; it was by the force of the exploding gas which had been generated within the body, which had been "found drowned." Persons may have been put in a coffin before they were perfectly dead, but it is absurd to suppose that life is possible after an interval of perfect seclusion from fresh air from the time of fastening the lid until the coffin reaches its last resting-place. The action of the gases in the cadaver will naturally and sufficiently explain all the appearances observed on occasions of opening the coffin after burial. The description which Hippocrates, the "Father of Medicine," gave of death over two thousand years ago, has never been improved upon. "The forehead wrinkled and dry; the eye sunken; the nose pointed, and bordered with a violet or black circle; the temples sunken, hollow, and retired; the lips hanging down; the cheeks sunken; the chin wrinkled and hard; the color of the skin leaden or violet; the hairs of the nose and eyelashes sprinkled with a yellowish white dust." This is as to the face; and when all observed, we may know that that face can never be lighted up to life again. But there are other proofs which do not leave the shadow of a doubt, as when the heart ceases to beat; the skin is pale and cold; a film is over the eye; the joints, first rigid, have become flexible; and a dark greenish color begins to form about the skin of the abdomen, the infallible sign of beginning corruption. But as we would have it done to us as the last request, let us with the utmost willingness allow the poor helpless, unresisting frame remain at least forty-eight hours under the unfastened lid after the surest proof of all has been noticed, the cessation of all movement of the chest and abdomen, for then the breath of life has gone out forever. The moments immediately preceding death from disease are probably those of utter insensibility to all pain, or of a delightful passivity, from that universal relaxation of every thing which pertains to the physical condition. Hence Louis XIV. is reported to have died saying: "I thought dying had been more difficult." The greatest surgeon of all ages, William Hunter, while dying said: "If this be dying, it is a pleasant thing to die." Dear reader, may you and I so live, that in the practice of bodily temperances and moral purities, death may be to us the gate of endless joy and sinless bliss.

